# Evaluating the Feasibility and Acceptance of a Mobile Clinical Decision Support System in a Resource-Limited Country: Exploratory Study

**DOI:** 10.2196/48946

**Published:** 2023-10-10

**Authors:** Kagiso Ndlovu, Nate Stein, Ruth Gaopelo, Michael Annechino, Mmoloki C Molwantwa, Mosadikhumo Monkge, Amy Forrestel, Victoria L Williams

**Affiliations:** 1 Department of Computer Science University of Botswana Gaborone Botswana; 2 Department of Product Management, VisualDx Rochester, NY United States; 3 Department of Business Development, Unleash Rochester, NY United States; 4 Department of Medical Education, Faculty of Medicine, University of Botswana Gaborone Botswana; 5 Department of Pediatrics and Adolescent Health, Princess Marina Hospital Gaborone Botswana; 6 Department of Dermatology, University of Pennsylvania Philadelphia, PA United States

**Keywords:** VisualDx, eHealth, technology acceptance model, clinical decision support, Botswana, dermatology, mobile phone

## Abstract

**Background:**

In resource-limited countries, access to specialized health care services such as dermatology is limited. Clinical decision support systems (CDSSs) offer innovative solutions to address this challenge. However, the implementation of CDSSs is commonly associated with unique challenges. VisualDx—an exemplar CDSS—was recently implemented in Botswana to provide reference materials in support of the diagnosis and management of dermatological conditions. To inform the sustainable implementation of VisualDx in Botswana, it is important to evaluate the intended users’ perceptions about the technology.

**Objective:**

This study aims to determine health care workers’ acceptance of VisualDx to gauge the feasibility of future adoption in Botswana and other similar health care systems.

**Methods:**

The study’s design was informed by constructs of the Technology Acceptance Model. An explanatory, sequential, mixed methods study involving surveys and semistructured interviews was conducted. The REDCap (Research Electronic Data Capture; Vanderbilt University) platform supported web-based data capture from March 2021 through August 2021. In total, 28 health care workers participated in the study. Descriptive statistics were generated and analyzed using Excel (Microsoft Corp), and thematic analysis of interview transcripts was performed using Delve software.

**Results:**

All survey respondents (N=28) expressed interest in using mobile health technology to support their work. Before VisualDx, participants referenced textbooks, journal articles, and Google search engines. Overall, participants’ survey responses showed their confidence in VisualDx (18/19, 95%); however, some barriers were noted. Frequently used VisualDx features included generating a differential diagnosis through manual entry of patient symptoms (330/681, 48.5% of total uses) or using the artificial intelligence feature to analyze skin conditions (150/681, 22% of total uses). Overall, 61% (17/28) of the survey respondents were also interviewed, and 4 thematic areas were derived.

**Conclusions:**

Participants’ responses indicated their willingness to accept VisualDx. The ability to access information quickly without internet connection is crucial in resource-constrained environments. Selected enhancements to VisualDx may further increase its feasibility in Botswana. Study findings can serve as the basis for improving future CDSS studies and innovations in Botswana and similar resource-limited countries.

## Introduction

In recent years, clinical decision support systems (CDSSs) have become increasingly popular [1]. This trend is partly spurred by the current COVID-19 pandemic requiring effective and efficient use of clinical data to inform strategic decision-making. Globally, health care systems are beginning to embrace CDSSs to augment the limited health human resource while reducing physical contact with physicians where possible. CDSS, defined as “information communication technologies (ICTs) that provide health care workers (HCWs) and patients with situation-specific advice that can inform their decision making,*”* [2] are popular in high-income countries and are increasingly becoming popular in resource-limited countries [3]. Botswana is a resource-limited country in sub-Saharan Africa that has embraced eHealth*—“*the use of ICTs for health*”* [4]—as a means toward (1) improving access to health care and provision of equitable health care in remote facilities, (2) achieving better customer satisfaction and improved patient outcomes and quality of care, (3) providing quick access to health information across the entire health sector, and (4) improving monitoring and evaluation of health care services [5].

Over the years, Botswana has been successful in achieving several health-related millennium development goals by reducing mortality among children aged <5 years, reducing the spread of HIV, reversing the incidence of malaria and other major diseases, and improving access to safe drinking water and basic sanitation [6]. Although less severe than elsewhere in sub-Saharan Africa, Botswana has a recognized inadequacy of health human resources, most significantly in primary health care [7]. Low physician to patient ratio (3.8:10,000) and nurse to patient ratio (32.6:10,000) are also reported [8]. Access to dermatological services continues to be one of the major challenges across the public and private sectors in Botswana, with most available services clustered in the capital city of Gaborone [9,10]. The number of dermatology specialist providers in Botswana’s public health care system has varied from none to, most recently, 2 full-time, Ministry of Health (MOH) employees and 3 contract specialists from Cuba. However, the demand for dermatology care in Botswana continues to be much higher than that can be provided by the current dermatology specialists, and waiting times for appointments can be ≥6 months [10]. There is a high prevalence of HIV and AIDS in Botswana, which results in increased demand for treatment of skin diseases [10]. Consequently, the MOH has provided care to patients with skin diseases through a combination of providers who lack sufficient training and reference materials at the point of care to help with the diagnosis and management of skin conditions seen in this environment. Most patients with skin complaints are initially examined at their local clinic by a general nurse or physician with little experience or knowledge about dermatology. Anecdotal evidence has shown that without access to dermatology treatment guidelines or reference material for dermatology, HCWs are rarely able to adequately address patients’ skin concerns. Consequently, this affects patients’ outcomes, as they may be given no diagnosis or treatment, given the wrong diagnosis and treatment, or referred directly to dermatology and required to wait until their appointment to receive help. They may be trialed on a plethora of indiscriminate treatments to address their skin concerns. This can result in wasted time and money; exposure to unnecessary side effects; and in some cases, significant morbidity, particularly in the case of skin cancers that can go undiagnosed and grow to an untreatable size before the patient reaches dermatology [11].

The shortage of dermatology specialists in Botswana necessitates efficient use of the limited resources and continuous empowerment of those commonly engaged in the management of prevalent skin conditions [12]. This suggests a critical need for a CDSS to ameliorate the current challenges within dermatology and other subspecialties in Botswana. Previous studies have demonstrated the promise of mobile-based CDSS in dermatology with varying uses within the health sector [13-16]. VisualDx is among the many platforms that could contribute to increased provider confidence and reduction in diagnostic errors in primary care settings [17,18]. VisualDx was implemented in Botswana to provide reference materials in support of diagnosis and management of dermatological conditions. VisualDx uses machine learning models, allowing nonspecialist providers to build custom differential diagnosis with patient-specific findings, view images highlighting variation in disease presentation, and view treatment recommendations, among other features; it has also been previously reported to have the potential to enhance diagnostic accuracy, aid therapeutic decisions, and improve patient safety [19].

Notwithstanding the potential benefits of VisualDx, previous studies have demonstrated that the implementation of any eHealth system is commonly associated with challenges [20,21]. There were limited previous data to inform the implementation of VisualDx as a CDSS to support dermatology services in Botswana. As such, a better understanding of the feasibility and acceptance of the tool by HCWs was needed to inform the ways of adapting it to the Botswana context and to guide sustainable implementation approaches across the health sector. A review of technology acceptance and adoption models by Taherdoost [22] identified several models explaining user adoption of new technologies and presenting factors that can affect user acceptance of the technology. A recent study in the United Arab Emirates used the extended Technology Acceptance Model (TAM) to explore the critical success factors for implementing artificial intelligence projects in the health sector [23]. Similarly, previous studies have identified the TAM as being prominent among key theoretical approaches used to understand people’s intentions to accept various forms of ICTs [24-26]. In essence, most studies that focus on explaining end user acceptance and predicting successful adoption of eHealth by health care organizations use the TAM as a basis [25-28]. The TAM centers on three belief constructs that have been found to significantly influence an individual’s (1) acceptance of (intention to engage in) a technology, (2) perceived usefulness, and (3) perceived ease of use [29]. It contends that a relationship exists between one’s intention to use technology and their actual use behavior [28,29].

Considering the novel use of the VisualDx CDSS in Botswana for dermatological services and the need to evaluate its feasibility, this study aimed to use the TAM to determine the acceptance of VisualDx and inform future adoption or adaptation strategies to minimize future implementation challenges. Study findings can assist in informing policy decisions and next steps toward a national rollout of VisualDx in Botswana and similar resource-limited countries.

## Methods

### Study Participants

Purposive sampling was used to select the study participants. Therefore, HCWs supporting dermatology clinics and medical students participating in dermatology coursework or rotations at health facilities and universities across Botswana were sent an email and WhatsApp invitations to participate in the study through the eHealth Research Unit at the University of Botswana (UB). Consent forms were also provided via email to confirm participation. A total of 18 participants volunteered to participate in the study initially.

As COVID-19 restrictions were eased in Botswana, the Greater Gaborone District Health Management Team (DHMT) was engaged to recruit more HCWs meeting the inclusion criteria to participate for the remainder of the study duration. The DHMT is a local authority under the MOH tasked with overlooking the management and staffing of primary care clinics. An additional 10 participants were enrolled, with approximately 3 months remaining in the study period, resulting in a total of 28 participants enrolled from 20 sites (health care facilities and UB) in Botswana. Participants were based at 6 health districts (Greater Gaborone: 21/28, 75%; Greater Palapye: 1/28, 4%; Greater Phikwe: 2/28, 7%; Greater Francistown: 2/28, 7%; Maun: 1/28, 4%; and Chobe: 1/28, 4%). These locations were selected because dermatology services are offered in these districts and the sites offer a comprehensive geographical coverage for the health sector in Botswana.

The authors acknowledge the small sample size and attribute sample limitations in part to funding constraints on the project to provide mobile devices to participants and the participants’ willingness to use personal devices (mobile phones) throughout the project. Funding constraints also limited the scale of the recruiting effort. Furthermore, the COVID-19 pandemic and the resulting strain on the health care system in Botswana was also a factor in limiting interest, as health care providers were uncertain where they would be allocated and for how long and how much time they would be able to devote to participating in the study. As such, the sample selection was biased toward providers who were already interested in using mobile health (mHealth) tools in their daily work.

### VisualDx Use

All participants used personal smartphones or tablet devices to download and install the VisualDx mobile app, with account credentials provided by VisualDx. They were offered mobile data vouchers to assist with the cost of data for the mobile app download and subsequent use. Initial training with the original cohort of participants was conducted using the Zoom (Zoom Video Communications) platform upon joining the study. Those recruited through the DHMT attended an in-person training session at the UB eHealth Research Unit. Training sessions covered IT skills, demonstrations of VisualDx app features, and practical application of VisualDx to common dermatologic and general medical conditions seen in Botswana. All training sessions were recorded and provided to participants who were unable to attend on the training day. Throughout the study duration, 6 case-based training sessions were provided to demonstrate the successful use of VisualDx to guide the clinical reasoning process.

Participants used VisualDx at their own discretion throughout the study period. A WhatsApp group was created to offer a platform for sharing announcements and seeking support related to the study.

### VisualDx CDSS Features

The VisualDx CDSS was developed by VisualDx and designed to be used on a mobile phone or tablet running an Android operating system (Google Inc) or iPhone operating system (Apple Inc). For the purposes of this study, the system was used for decision support in varying settings to reduce diagnostic errors and suggest management options for dermatological and other conditions. Offline capability was recently developed for Android devices and used to further increase the potential utility of VisualDx in areas with limited internet connectivity.

**Figure 1:**
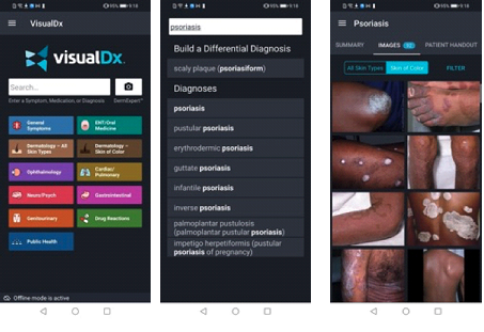
Screenshots of the clinical decision support system features—searching for a diagnosis to view images and detailed diagnosis information.

**Figure 2:**
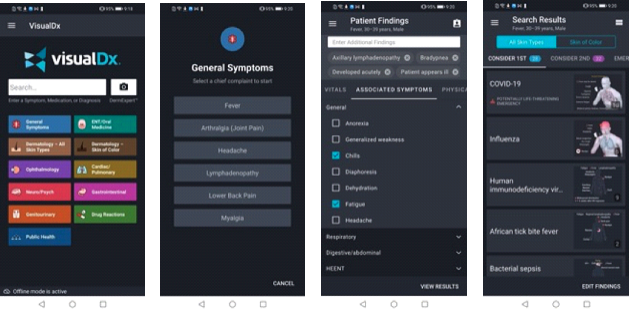
Screenshots of the clinical decision support system features—building a differential diagnosis based on the patient’s symptoms.

**Figure 3:**
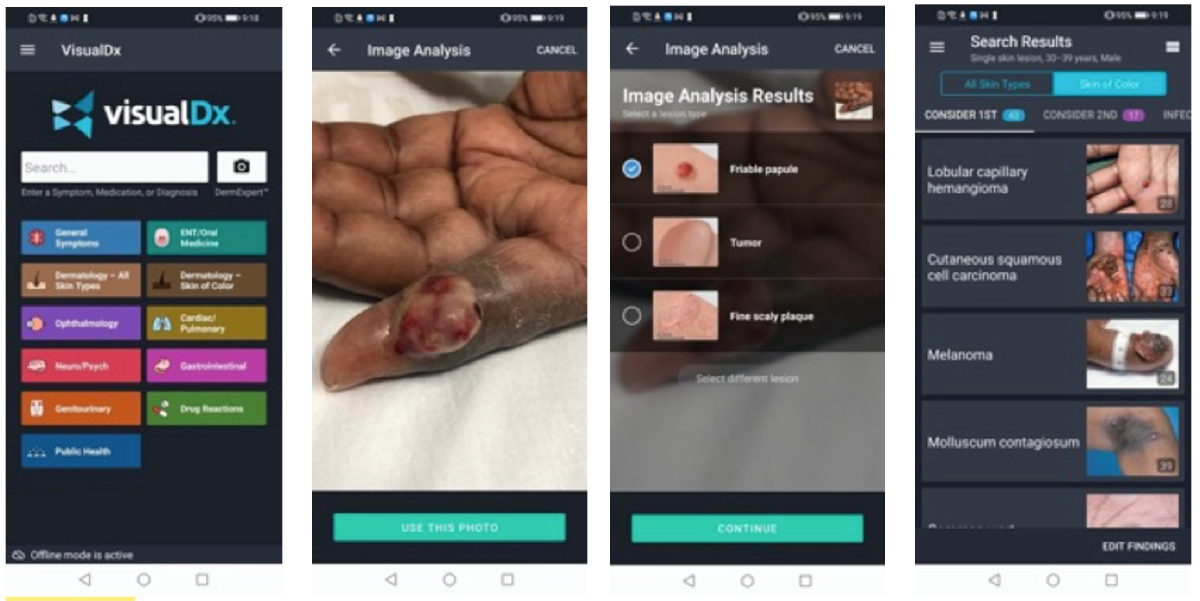
Screenshots of the clinical decision support system features—using VisualDx’s DermExpert feature to analyze a skin problem with artificial intelligence.

VisualDx features used include (1) searching directly for any of >4000 diseases for clinical information including therapy options, appropriate tests, and management pearls (Figure 1); (2) building a custom differential diagnosis based on chief complaints across all fields of medicine (Figure 2); and (3) taking photos of a skin condition, and through in-built artificial intelligence techniques, receiving a list of possible conditions matching the images provided (Figure 3).

### Data Collection

An explanatory, sequential, mixed methods design [30] was used to assess the feasibility and acceptance of VisualDx as a CDSS tool in Botswana. Quantitative data were collected from a series of 3 surveys delivered at the beginning, middle, and end of the study. The survey was designed in 3 parts to assess any changes over time in participants’ acceptance as they became familiar with the system. RG and NS created the first draft of the survey questions and the interview script. Survey questions were then reviewed and edited by all authors to avoid noted ambiguities. After the survey questions were configured in the REDCap (Research Electronic Data Capture; Vanderbilt University) system [31], pretesting of the surveys was conducted by RG, NS, and KN before being enhanced through improved branching logic.

VisualDx mobile app use data were also collected from the VisualDx servers through existing event tracking mechanisms. Qualitative data from semistructured interviews were collected (one 30- to 60-min interview with each participant). Both survey and interview tools were used by the authors to meet the study objective.

All surveys were administered through REDCap, with links provided to the participants for access on their personal or work devices. REDCap is a secure (Health Insurance Portability and Accountability Act and General Data Protection Regulation compliant) system for supporting electronic data capture for research and operational support projects. The first survey was distributed to participants immediately following their initial mobile app training (March 2021). The second survey was delivered in the third month of the study period (May 2021). For the cohort of participants that started midway through the study, this survey was delivered after 1 month of participation (July 2021). The final survey was completed at the end of the study period (August 2021). All 3 surveys were offered to the same participants, but owing to their conflicting work schedules, not all were able to participate. Surveys had closed-ended questions (dichotomous, multiple-choice, and Likert-scale questions) and open-ended questions. The prepilot survey had 4 dichotomous questions, 8 multiple-choice questions, a 5-point Likert-scale question related to participants’ comfort in diagnosis using VisualDx (ordinal scale: 1=no interest, 2=little interest, 3=neutral, 4=interested, and 5=very interested), and 8 open-ended questions. The midpilot survey had 4 dichotomous questions, 15 multiple-choice questions, a 5-point Likert-scale related question to rating participants’ comfort while using VisualDx (ordinal scale: 1=not comfortable at all, 2=somewhat uncomfortable, 3=neutral, 4=comfortable, and 5=very comfortable), and 8 open-ended questions. The postpilot survey had 3 dichotomous questions, 19 multiple-choice questions, a 5-point Likert-scale question related to rating VisualDx’s relevance to their work (ordinal scale: 1=irrelevant, 2=somewhat relevant, 3=neutral, 4=relevant, and 5=very relevant), and 7 open-ended questions.

In June 2021 (three months into the study period), participants were contacted individually via WhatsApp to schedule semistructured interviews to gain more in-depth knowledge regarding the participants’ survey responses. All interviews were conducted remotely via Zoom platform, with each participant providing verbal consent to record. Interview recordings were then transcribed verbatim and reviewed by all researchers.

SQL statements were executed against the VisualDx database to obtain use data associated with the study participants’ user accounts.

### Data Analysis

Quantitative data were summarized using descriptive statistics, and the mean and median were calculated using the REDCap system. Interview transcripts were uploaded to the Delve software for coding. Qualitative interview data were analyzed using the widely accepted principles of thematic analysis by Terry et al [32] to categorize data into key themes, which were later aligned to TAM constructs. Iterative transcript review and deductive coding [33] were performed independently by NS, RG, and M Molwantwa. A predefined list of descriptive codes was developed and later discussed by all authors: “clinical decision support,” “eHealth,” “ease of use or usability,” “continuing education,” “Internet connectivity,” “electronic health record,” “national implementation feasibility,” “technology acceptance,” “usage facilitators,” and “usage barriers.” Subcodes were created to further categorize the interview responses in more detail (shown in Multimedia Appendix 1). The codes were later grouped into 4 broad themes: “governance,” “technology infrastructure,” “human resource capacity development,” and “usability.”

Use data associated with the study participants’ user accounts were analyzed in Excel to generate basic descriptive statistics related to the frequency of use, mode of access, and most commonly used features.

The surveys, interviews, and use data analysis were guided by the TAM constructs of perceived acceptance, usefulness, and ease of use of the VisualDx CDSS.

### Ethical Considerations

The study protocol was approved by UB’s institutional review board (UBR/RES/IRB/BIO/223) and the Botswana Ministry of Health and Wellness (Health Policy, Development, Monitoring and Evaluation: 13/18/1) in December 2020. The approved protocol was implemented over 6 months from March 2021 through August 2021. All study participants provided informed consent electronically.

## Results

### Overview

**Figure 4:**
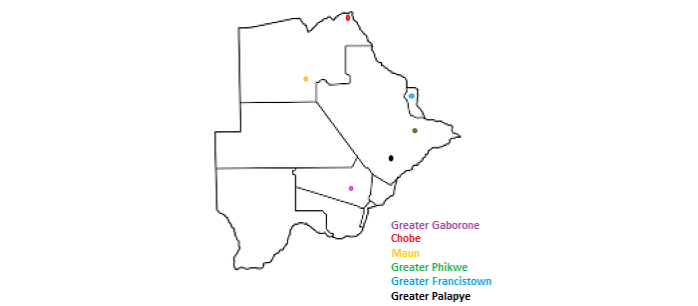
Geographical representation of the study sites.

Of the 28 study participants, 9 (32%) were aged between 20 and 29 years, 14 (50%) were aged between 30 and 39 years, 4 (14%) were aged between 40 and 49 years, and 1 (4%) did not specify their age range. Of the 28 participants, 12 (43%) were physicians, 12 (43%) were nurses, and 4 (14%) were medical students. Of the 28 participants, 14 (50%) specified their primary place of work as an outpatient clinic, 10 (36%) worked in a primary hospital, 1 (4%) worked in both clinics and hospitals, and 3 (11%) did not specify. Of the 28 participants, 20 (71%) practiced general or family medicine, 2 (7%) specialized in dermatology, 2 (7%) specialized in pediatrics, and 4 (14%) practiced another medical specialty. Multimedia Appendix 2 and Figure 4 show the total number of study participants per location and the geographical representation of study sites on the Botswana map, respectively.

**Figure 5:**
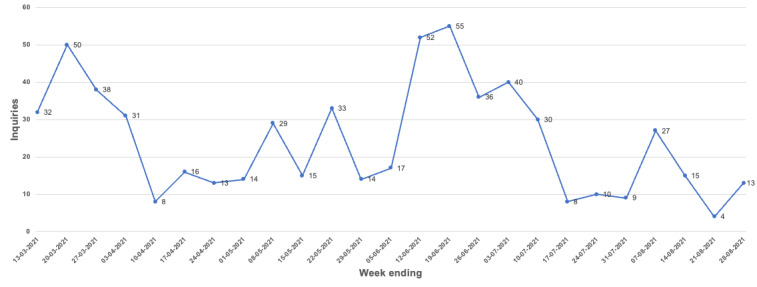
VisualDx weekly use summary.

**Figure 6:**
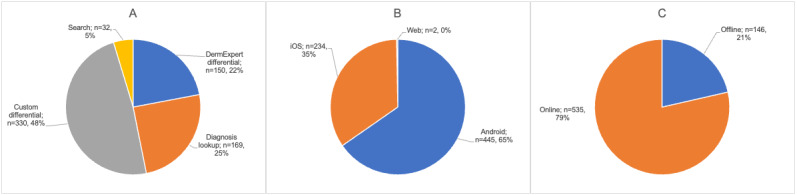
VisualDx use summary categorized according to (A) use case, (B) operating system, and (C) connectivity mode.

Of the 28 participants, 7 (25%) successfully downloaded and used VisualDx in offline mode. This includes those participants who did not have access to offline mode (ie, participants using iOS devices). Of the participants who had access to offline functionality, 28% (7/25) were able to successfully download and use VisualDx’s offline features. Figures 5 and 6 show the weekly use summary and use categorized according to operating system, type of use case, and offline and web-based use, respectively.

### Surveys

#### Pre–VisualDx Pilot Survey Responses

The first survey was distributed before the pilot began in March 2021 and had a 100% (28/28) response rate. Questions were primarily focused on participants’ interest level in eHealth technology, their familiarity with the VisualDx platform, and existing tools they currently used for the diagnosis and treatment of dermatological conditions (Table 1).

Overall, study participants showed interest in using mHealth or eHealth technologies to support their daily work. This was demonstrated by the high scores recorded (1=not interested at all to 5=very interested) when asked to rate their level of interest in using mHealth technologies.

**Table 1:** Tools used by participants before VisualDx (N=28).

Tools currently used for clinical decision support			Participants, n (%)
Textbooks			23 (82)
Journal articles			11 (39)
Google search engine			24 (86)
**Other**			
	Medscape	3 (11)	
	Uptodate	3 (11)	
	Accessmedicine	1 (4)	
	AMBOSS	1 (4)	
	Osmosis	1 (4)	
	VisualDx	1 (4)	
	U-central	1 (4)	
	EMGuidance	1 (4)	
	Merck manuals	1 (4)	
	Program guidelines and protocols	1 (4)	

#### Mid–VisualDx Pilot Survey Responses

The second survey was distributed in early June 2021 and had a 79% (22/28) response rate. The survey identified participants’ barriers to using VisualDx and their perceived ease of use and usefulness of the VisualDx platform (Table 2).

During the midpilot survey, VisualDx was used every day by 9% (2/22), a few times a week by 59% (13/22), a few times a month by 18% (4/22), or once every couple of months by 14% (3/22) of participants. Of the 22 survey respondents, 15 (68%) used VisualDx during a patient encounter, 11 (50%) used it immediately before or after a patient encounter, 18 (82%) used it as a studying or educational tool outside work, and 2 (9%) used it for other purposes.

Of the 22 survey respondents, 15 (68%) indicated that they have not encountered any issues or barriers when trying to use VisualDx, whereas 7 (32%) highlighted the following barriers:

1. *Lack of reliable internet* (4/22, 18%)
2. *I find VisualDx difficult to use* (1/22, 5%)
3. *I could not find a good time to use VisualDx in my clinical workflow* (4/22, 18%)
4. *I do not trust the results or content in VisualDx* (1/22, 5%)

**Table 2:** Midpilot perceived ease of use and perceived usefulness (Technology Acceptance Model [TAM]–related elements from the midpilot survey).

TAM construct	Question	Likert scale	Median	Mode
Perceived ease of use	How easy is it to use VisualDx?	1=very difficult to 5=very easy	4.5	5
Perceived usefulness	Rate the quality and relevance of the diagnosis content (text and images) in VisualDx.	1=very irrelevant to 5=very relevant	4.5	5

Survey respondents further reported VisualDx to be useful in the following ways: (1) changing a patient’s treatment plan based on information in VisualDx (7/19, 37%), (2) diagnosing or treating a patient (14/19, 74%), (3) educating a patient about their condition or sharing photos with them (10/19, 53%), and (4) confirming a suspected diagnosis (11/19, 58%). Three survey respondents skipped the question related to useful applications of VisualDx.

Other useful scenarios for VisualDx obtained from the midpilot survey are noted in Multimedia Appendix 3.

#### Post–VisualDx Pilot Survey Responses

The final survey was distributed at the end of August 2021 and had 68% (19/28) response rate. Some questions from the second survey were repeated to assess change over time, and additional 1 to 5 Likert-scale questions and dichotomous questions were asked to assess participants’ perceptions about VisualDx. These were aligned to the TAM constructs (Table 3).

The third survey showed that among the 19 participants, VisualDx was used every day by 1 (5%), a few times a week by 10 (53%), a few times a month by 4 (21%), or once every couple of months by 3 (16%), and 1 (5%) participant rarely or never used VisualDx.

The third survey highlighted that among the 19 respondents, 8 (42%) did not encounter any issues or barriers when trying to use VisualDx, whereas 11 (58%) reported the following barriers while trying to use VisualDx:

1. *Lack of reliable internet* (7/19, 37%)
2. *I find VisualDx difficult to use* (1/19, 5%)
3. *I could not find a good time to use VisualDx in my clinical workflow* (4/19, 21%)
4. *Other barriers* (2/19, 11%)

Of the 19 survey respondents, 18 (95%) found “VisualDx easy to access and easy to use,” whereas 1 (5%) disagreed with the statement.

Perceived usefulness of the VisualDx platform was further highlighted during the third and final survey, as summarized in Tables 4 and 5.

At the end of the study period, participants demonstrated overall acceptance of the VisualDx platform (Table 6).

**Table 3:** VisualDx’s perceived ease of use, usefulness, and acceptance during the postpilot survey (Technology Acceptance Model [TAM]–related elements from the postpilot survey).

TAM construct	Question	Likert scale	Median	Mode
Perceived ease of use	How easy is it to use VisualDx?	1=very difficult to 5=very easy	5	5
Perceived usefulness	Rate the quality and relevance of the medical content in VisualDx.	1=not relevant at all to 5=very relevant	4	5
Acceptance	How would you rate your comfort level with diagnosis and treating dermatology issues?	1=not comfortable at all to 5=very comfortable	4	3

**Table 4:** VisualDx’s perceived usefulness during the postpilot survey (agree or disagree or neither agree nor disagree; Technology Acceptance Model–related elements from the postpilot survey; n=19).

Question (multiple choice)	Agree, n (%)	Disagree, n (%)	Neither, n (%)
I feel that the diagnosis content and images in VisualDx are relevant to my practice.	18 (95)	0 (0)	1 (5)
VisualDx has helped me educate patients and build patient trust.	16 (84)	0 (0)	3 (16)
VisualDx does not go far enough in recommending next steps.	3 (16)	15 (79)	1 (5)

**Table 5:** VisualDx’s perceived usefulness scenarios during the postpilot survey (Technology Acceptance Model–related elements from the postpilot survey; n=19).

Question (multiple choice)	Yes, n (%)	No, n (%)	Not sure, n (%)
Have you encountered any scenarios where VisualDx provided a clear benefit to you or your patient?	16 (84)	3 (16)	0 (0)
Do you feel that the information you gain from VisualDx helps you to make more accurate diagnosis?	17 (90)	0 (0)	2 (11)
Has VisualDx made your clinician work easier?	17 (90)	0 (0)	2 (11)
Has VisualDx helped you diagnose and manage skin disease?	18 (95)	0 (0)	1 (5)
Has VisualDx improved your ability to manage non-dermatologic conditions?	15 (79)	1 (5)	3 (16)

**Table 6:** VisualDx’s perceived acceptance (agree or disagree or neither agree nor disagree; Technology Acceptance Model–related elements from the postpilot survey; n=19).

Question (multiple choice)	Agree, n (%)	Disagree, n (%)	Neither, n (%)
I feel more confident in my work knowing that I have VisualDx available as a reference tool.	18 (95)	1 (5)	0 (0)
I found it challenging to find the right time in my clinical workflow to use VisualDx.	10 (53)	5 (26)	4 (21)
I will continue to use VisualDx in my day-to-day work.	19 (100)	0 (0)	0 (0)

### Interviews

Of the 28 participants, 17 (61%) participated in web-based interview sessions conducted at different times throughout the study period. Interviewees described their daily work, challenges encountered on a regular basis, and overall experiences with or perceptions about VisualDx. Participants’ responses were categorized into 4 themes (governance, infrastructure, human resource capacity development, and usability), which highlighted the possible factors that could affect the sustainable implementation of the VisualDx platform in Botswana (Table 7).

**Table 7:** Thematic presentation of factors affecting the sustainable implementation of VisualDx (themes deduced from the survey’s open-ended questions and interviews).

Themes	Example quotes
Governance	“There’s a lot of lack of resources in Botswana. So we couldn’t necessarily do all the tests that they recommended. And in terms of management, a lot of the medication that they recommend is not exactly here, we need to find something similar in that drug class.” “I think this is a great idea and innovation. So my thinking is...if all doctors can be able to get the app and use it, it will make a difference. Because since I got the VisualDx, I don’t remember the last time I pulled in experts to ask about a skin problem. I don’t remember. So I think it can make a difference.”
Technology infrastructure	“When I was downloading it - when we were in UB - it was taking time to download. I think it was the network issues but I managed to download [VisualDx offline content]. But then I think it was within maybe 30 to 45 minutes or so.” “My other problem is my camera...when someone presents with a skin condition I got a problem to take a photo because of my camera.”
Human resource capacity development	“You know, I’m not an electronic oriented person.” “So for me, things like VisualDx is a way to keep refreshing my brain as a practicing doctor as to the things that I may have forgotten because unfortunately in Botswana we only have ourselves sometimes to depend on, especially in the district other than the call away physician.”
Usability	“...When you look into management of certain cases, for example, if I look into B12 deficiency and then it gives you management, it’s not very precise in how you have to manage it in terms of dosage, or the frequency of doing blood tests and what not. And I felt that was a bit lacking and in management section for a couple of things that I looked up.” “So when you see a case, like the first three suggestions, it doesn’t speak to our population.” “And once more, it is easy and friendly to use in setups where you are consulting many patients, it doesn’t take too long to use the tool.” “You can even get the answers right there with the patient.” “So it’s really useful to have offline. And it’s advisable in Botswana to always have for all applications an offline mode.” “And you get more specific diagnosis, I do not know if specific is the right word, but maybe tailored diagnosis like more towards what you are looking for, especially with the skin of colour option, it really made a huge difference for me.” “[VisualDx] is phenomenal, I love it. And it has just really made a huge difference.” “I think it’s an excellent app. Especially for the dermatological cases.”

## Discussion

### Principal Findings

Overall, this study demonstrated the potential for the acceptance of the VisualDx platform by HCWs in Botswana. All participants in the initial survey (28/28, 100%) expressed interest in using mHealth or eHealth technology to support their daily work. The willingness to use or learn about mHealth was previously identified as an important factor toward technology acceptance [34] in addition to perceived ease of use and perceived usefulness, as outlined in the TAM. Most responses showed positive statements toward TAM constructs (Table 2: 22/28, 79%; mode 5 and Table 4: 19/28, 68%; mode 5). On the basis of our findings, successful acceptance of the VisualDx platform by HCWs in Botswana was achieved; however, other factors that could influence the acceptance of VisualDx were noted and organized into themes—governance, technology infrastructure, human resource capacity development, and usability (Table 7). These are essential for influencing technology acceptance and highlight an organization’s readiness to adopt or adapt a new technology. Similarly, previous studies have associated failure of eHealth system implementations with the lack of eHealth readiness (the preparedness of health care institutions or communities for the anticipated change brought by programs related to information and communications technology) [21,34-39]. Moreover, constraints to the adoption of eHealth in Africa have been previously reported by the World Health Organization to include low ICT budgets, poor infrastructure for communication, erratic electricity supply, and inadequate human resource capacity [40], all of which are reflections of lack of readiness.

Barriers to the implementation of the VisualDx CDSS were reported and aligned to the identified themes. Some of the barriers encountered when trying to use VisualDx are consistent with those found in previous studies of mobile CDSS (MCDSS), including the perceived irrelevance of information by some participants, lack of technical skill or savvy, and lack of access to technology or internet connectivity [41,42]. A recent study by Zakerabasali et al [39] also highlighted the importance of understanding barriers to the adoption of mHealth apps among providers and engaging them in the adoption process, as that is essential for their successful implementation.

In essence, the acceptance of VisualDx CDSS can be greatly influenced by its perceived ease of use and perceived usefulness. These factors have been previously documented as positive influences toward technology adoption [25,43].

### Perceived Ease of Use

Results related to the perceived ease of use of VisualDx CDSS were largely positive, as evidenced by the modal score of 5 out of 5 on the Likert scale indicating “very easy to use” on the second and third surveys (Tables 2 and 3).

Most interviews highlighted that VisualDx is easy to use owing to user-friendly interfaces enabling quick information retrieval at the point of care. The importance of supporting user-friendly interfaces with real-time feedback and decision support capabilities in mHealth solutions was also highlighted in previous studies [44-46].

Despite the reported perceived ease of use, among the 17 interviewees, 4 (24%) cited usability concerns about the VisualDx platform suggesting “Differential diagnosis results as too broad/not enough recommendation of next steps”:

When you look into management of certain cases, for example, if I look into B12 deficiency and then it gives you management, it’s not very precise in how you have to manage it in terms of dosage, or the frequency of doing blood tests and what not. And I felt that was a bit lacking and in management section for a couple of things that I looked up.

Other interviewees (3/17, 18%) reported “Perceived lack of relevant information in the app”:

So when you see a case, like the first three suggestions, it doesn’t speak to our population.

Barriers to using VisualDx including the lack of technical savvy and lack of reliable internet access were also reported, suggesting the need to strengthen health human resource capacity development and the provision of adequate technology infrastructure. This further suggests an ongoing need for eHealth providers to tailor solutions to the contexts in which they are being delivered, including medical content, the delivery of training resources, and offline access. These measures were also previously suggested in dealing with inadequate technology infrastructure and lack of skilled personnel to support mHealth interventions [25,41,46]. Considering that VisualDx is designed to be adaptable to specific country context, participants’ responses could directly influence further improvements to the medical content and features of the platform. Overall, responses related to the *ease of use* construct were positive (Tables 3 and 4).

### Perceived Usefulness

Study participants identified the VisualDx CDSS to be useful overall, highlighting its potential to influence multiple areas. The range of features available in VisualDx lent to its perceived usefulness across the study population, as different providers found the app to be useful for different situations (during a patient encounter, immediately before or after a patient encounter, and as a studying or educational tool outside work). The perceived relevance of VisualDx’s medical content and the convenience of mobile and offline access are consistent with previous MCDSS studies [44-46].

VisualDx was perceived to be particularly useful in supporting point-of-care decision-making, patient outcomes and engagement, access even when there is unreliable connectivity, reduction in referrals to specialists, and continuing medical education and professional development.

By the end of the study, 89% (17/19) of the survey respondents reported that VisualDx generally helped them make more accurate diagnosis, and 95% (18/19) indicated that VisualDx helped them diagnose and manage skin disease specifically (Table 5). These findings emphasize the perceived usefulness of the VisualDx CDSS and its relevance toward supporting diagnosis across all areas of medicine, especially dermatological conditions.

Contrary to a recent study where patients highlighted lack of confidence in the mHealth system [45], VisualDx was considered to be useful for educating patients and building patients’ trust during encounters. Overall, 84% (16/19) of the surveyed participants in the postpilot survey indicated that they had encountered a situation in which using VisualDx provided a clear benefit, and 84% (16/19) also indicated that VisualDx helped to educate patients and build patient trust throughout encounters (Tables 4 and 5). The increased user confidence in the VisualDx CDSS could also be a result of some measures put in place by VisualDx Corporation to ensure data privacy and confidentiality. Notably, VisualDx collects only anonymized and generalized demographic information about the patient to provide a differential diagnosis. Even when using the “DermExpert” artificial intelligence tool, the image of the patient remains on the device at all times and is discarded immediately after the analysis is complete. This alleviates any data security concerns and allows the tool to conform to data protection standards such as the Health Insurance Portability and Accountability Act [47], General Data Protection Regulation [48], and Botswana Data Protection Act of 2018 [49].

Of all VisualDx use throughout the study, 21.4% (146/681 uses) was in offline mode (Figure 6). Removing iOS users from the analysis (as iOS users did not have access to offline mode) shows 32.8% (146/445 uses) of use in offline mode by Android users only. This suggests that most users encounter situations of limited connectivity regularly and choose to use VisualDx offline as a mitigation. Notably, however, users still need internet connection to complete the download of offline content. Overall, 82% (14/17) of the interviewed participants said that the lack of reliable internet was a barrier to using the VisualDx CDSS (Table 7). Kabukye et al [50] highlighted the need to address inadequate computer infrastructures challenges before implementation of any electronic health record systems.

mHealth tools were previously reported to have the potential to upskill nonspecialist HCWs, allowing them to address more issues than they otherwise might not be able to address without specialist guidance [46]. By the end of the study, among the 19 participants, case referrals to a specialist or another provider were reported less than once per week by 8 (42%), 1 to 3 times per week by 6 (32%), 3 to 6 times per week by 3 (16%), and ≥7 times per week by 2 (11%) participants. At the beginning of the study, the modal response for frequency of referrals was 1 to 3 times per week (14/27, 52%), whereas at the end of the study, after using VisualDx, the modal response to the same question was less than once per week (8/19, 42%). This is of particular significance in Botswana, where the health care system is experiencing a shortage of medical specialists and especially dermatologists [9,10].

Most survey respondents (16/19, 84%) used VisualDx outside their work as a studying or educational tool. In addition, 24% (4/17) of the interviewed participants expressed that using VisualDx allowed them to stay up to date on the latest best medical practices and challenge the way that they have handled certain conditions in the past. The ability to keep users’ knowledge and skills fresh by using an eHealth application is a facilitator of use.

Actual use statistics helped to more specifically identify the situations in which participants found VisualDx to be the most useful. The use case for building a differential diagnosis accounted for 70.5% (480/681) of use, whereas 29.5% (201/681) of use came from participants searching directly for a specific condition. Of the differential diagnosis uses, 31.3% (150/480) were generated using the “DermExpert” machine learning algorithm, whereas 68.8% (330/480) were differentials generated by manually entering a custom set of symptoms (Figure 6). This use pattern suggests that users in Botswana perceive the differential diagnosis features of VisualDx to be the most useful, whether they are using the tool in situations of uncertainty, looking for a second opinion, or confirming that they are not missing any diagnostic possibilities. The relatively low use of the direct diagnosis search feature could suggest that users are well trained to handle the conditions that they are already familiar with or they have other tools or references (eg, MedScape, UpToDate, and others listed in Table 1) that they tend to access for this use case.

### Acceptance

Participants’ general acceptance of VisualDx as an MCDSS tool was confirmed by multiple data points. Of the 17 interviewed participants, 16 (94%) indicated positive overall user satisfaction (Multimedia Appendix 1), and 9 (53%) expressed the importance of a nationwide rollout of VisualDx across Botswana to help upskill the country’s general practitioner workforce and reduce stress on referral hospitals (Table 7). Furthermore, 100% (19/19) of the survey respondents indicated that they intend to continue using VisualDx after completion of the study (Table 6).

### Study Limitations

The study protocol required deviation from the initial plan primarily owing to restrictions and delays caused by the COVID-19 pandemic. Most notably, almost all training sessions and interviews had to be conducted remotely via the Zoom platform owing to restrictions on in-person gatherings. Researchers were unable to visit the clinics and facilities in person to provide support and further training. All outreach and follow-ups had to be completed through WhatsApp messenger.

In addition, participation was limited as COVID-19 resulted in some participants being reassigned to efforts such as vaccine distribution or other scenarios, which would not be applicable for VisualDx use. Moreover, compliance with completing surveys and scheduling interviews was not 100%; some participants failed to complete these key data collection end points. This was likely owing in part to the fact that almost all study activity was conducted remotely without in-person follow-up. Increased stress and workload owing to COVID-19 surges in Botswana also likely contributed to the lack of compliance.

### Future Direction

Consistent with the study findings, the authors have identified the following acceptance-related issues and associated mitigation strategies as essential to informing next steps.

Survey responses and themes from the interviews conducted pointed out areas of concern for widespread adoption such as the lack of locally specific guidance and content, lack of reliable internet connectivity (especially for iPhone users), uncertainty about when to use VisualDx in the clinical workflow, lack of technical savvy, and perception that VisualDx is only for dermatologic concerns.

At the individual level, TAM is based on the assumption that when users perceive that a type of technology is useful and easy to use, they will be willing to use it. However, that alone may not adequately address the issues raised. Beyond end users’ individual acceptance, there are models that also recognize the role of organizational readiness in enhancing successful acceptance and, consequently, successful adoption of the VisualDx platform. Organizational issues such as leadership buy-in, change management strategies, and alignment of eHealth initiatives to organizational eHealth mandate or vision have been previously identified as important drivers for technology adoption [50-54]. Moreover, seven leadership behaviors associated with successful outcomes in health IT adoption include (1) communicating clearly about visions and goals, (2) providing support, (3) establishing a governance structure, (4) establishing training, (5) identifying and appointing champions, (6) addressing work process change, and (7) following up [55]. A holistic strategy for rollout and adoption of mHealth tools such as VisualDx must account for both end user acceptance and these organizational and governance-related factors.

Further studies in the following areas may build upon the findings of this study to further inform mHealth adoption strategies in Botswana and other contexts:

1. Similar TAM studies with health care providers at different levels and in different contexts to identify where VisualDx or other mHealth tools are more or less likely to be accepted by end users
2. Studying the impact of VisualDx or other mHealth tools on patient outcomes—demonstrating a positive impact on patient outcomes could help to build leadership buy-in
3. Assessing the quality of the VisualDx app using the Mobile Application Rating Scale [56] to better understand the strengths and areas to improve

### Conclusions

VisualDx was a well-received MCDSS tool among the study population and has the potential to upskill and empower general practitioners to do more at the point of care. Through widespread use of VisualDx, it could be reasonably hypothesized that benefits such as improved patient outcomes, reduced stress on the medical system through reduced need for referrals, and improved continuing medical education could be realized. Barriers to the use of VisualDx were identified as potential areas of improvement for this and other mHealth tools targeting HCWs in Botswana and other countries with similarly developing health care systems. To ensure successful widespread adoption of an mHealth tool, end user acceptance must be paired with organizational readiness to fully embed the solution into the existing context.

## Appendix

Multimedia Appendix 1 Facilitators and barriers influencing VisualDx use identified in participant interviews, by themes.

Multimedia Appendix 2 Number of study participant per location or site.

Multimedia Appendix 3 VisualDx useful scenarios during the second survey.

## Abbreviations

CDSS: clinical decision support system
DHMT: District Health Management Team
HCW: health care worker
ICT: information communication technology
MCDSS: mobile clinical decision support system
mHealth: mobile health
MOH: Ministry of Health
REDCap: Research Electronic Data Capture
TAM: Technology Acceptance Model
UB: University of Botswana

## Data Availability

The data sets used and analyzed during the study are available from the corresponding author upon reasonable request.
