# Supplementary material for: Evaluating the Feasibility and Acceptance of a Mobile Clinical Decision Support System in a Resource-Limited Country: Exploratory Study
Source: JMIR Form Res. 2023 Oct 10;7:e48946. doi: 10.2196/48946 (PMC10599284; doi:10.2196/48946)
Supplement: Multimedia Appendix 1 [file formative_v7i1e48946_app1.pdf]

This is a Multimedia Appendix to a full manuscript published in the J Med Internet Res. For full copyright and citation information see http://dx.doi.org/10.2196/48946

Appendix 1: Facilitators and barriers influencing VisualDx usage identified in participant interviews, by themes.

**Theme**	**Barrier to using VisualDx**	**Facilitator for using VisualDx**
**Governance**	Lack of localized or region-specific guidance.<br>**4 (23.5%)**	Perceived nationwide demand for mHealth and VisualDx<br>**9 (52.9%)**
**Technology Infrastructure**	Lack of reliable internet connectivity or slow app performance.<br>**14 (82.4%)**<br><br>Lack of technical savvy, or using an old or broken device.<br>**3 (17.6%)**	Ability to access app offline<br>**3 (17.6%)**
**Human Resource Capacity Development**	Lack of technical savvy.<br>**3 (17.6%)**	Perceived educational benefit from using the app<br>**4 (23.5%)**<br><br>Lack of confidence or experience making diagnosis<br>**4 (23.5%)**
**Usability**	Differential diagnosis results too broad/not enough recommendation of next steps.<br>**4 (23.5%)**<br><br>Perceived lack of relevant information in the app. **3 (17.6%)**	Positive user satisfaction<br>**16 (94.1%)**<br><br>Ability to find information quickly at point of care<br>**5 (29.4%)**
