# Supplementary material for: Evaluating the Feasibility and Acceptance of a Mobile Clinical Decision Support System in a Resource-Limited Country: Exploratory Study
Source: JMIR Form Res. 2023 Oct 10;7:e48946. doi: 10.2196/48946 (PMC10599284; doi:10.2196/48946)
Supplement: Multimedia Appendix 2 [file formative_v7i1e48946_app2.pdf]

This is a Multimedia Appendix to a full manuscript published in the J Med Internet Res. For full copyright and citation information see http://dx.doi.org/10.2196/48946

Appendix 2: Number of study participant per location or site

**Number of participants**	**Locations**	**Mobile Device Operating System**
21 (75%)	Greater Gaborone area *(Princess Marina Referral Hospital, Siga Clinic, Lesetlhana Clinic, Nkoyaphiri Clinic, Old Naledi Clinic, G-West Clinic, Phase 2 Clinic, Block 8 Clinic,, Baylor clinic, Block 3 clinic, Hospital Way Medical Center Clinic, Otse Clinic, Scottish Livingstone Hospital)*	Android (20)<br>iOS (1)
2 (7.1%)	Greater Phikwe area *(Selibe Phikwe Government Hospital, Bobonong Primary Hospital,)*	Android (2)
2 (7.1%)	Greater Francistown area *(Nyangabwe Referral Hospital)*	Android (2)
1 (3.6%)	Maun *(Maun Clinic)*	iOS (1)
1 (3.6%)	Chobe *(Shakawe Clinic)*	Android (1)
1 (3.6%)	Greater Palapye area *(Mahalapye Primary Hospital)*	iOS (1)
