# Supplementary material for: Evaluating the Feasibility and Acceptance of a Mobile Clinical Decision Support System in a Resource-Limited Country: Exploratory Study
Source: JMIR Form Res. 2023 Oct 10;7:e48946. doi: 10.2196/48946 (PMC10599284; doi:10.2196/48946)
Supplement: Multimedia Appendix 3 [file formative_v7i1e48946_app3.pdf]

This is a Multimedia Appendix to a full manuscript published in the J Med Internet Res. For full copyright and citation information see http://dx.doi.org/10.2196/48946

Appendix 3: VisualDx useful scenarios during the second survey

Question	Yes	No	Not sure
Do you feel that the information you gain from VisualDx helps you make more accurate diagnosis?	18 (81.8%)	0 (0.0%)	4 (18.2%)
Has VisualDx made your clinical work easier?	18 (81.8%)	0 (0.0%)	4 (18.2%)
Has VisualDx helped you diagnose and manage skin disease?	20 (90.9%)	0 (0.0%)	2 (9.1%)
Has VisualDx helped you diagnose and manage non-dermatologic conditions?	14 (63.6%)	3 (13.6%)	5 (22.7%)
Have you encountered any scenarios where using VisualDx provided a clear benefit to you or your patient?	19 (86.4)	3 (13.6%)	0 (0.0%)
